# Shyness, Sport Engagement, and Internalizing Problems in Chinese Children: The Moderating Role of Class Sport Participation in a Multi-Level Model

**DOI:** 10.3390/bs14080661

**Published:** 2024-08-01

**Authors:** Rumei Zhao, Xiaoxue Kong, Mingxin Li, Xinyi Zhu, Jiyueyi Wang, Wan Ding, Xuechen Ding

**Affiliations:** 1 School of Psychology, Shanghai Normal University, Shanghai 200234, China; 1000546535@smail.shnu.edu.cn (R.Z.); 1000527473@smail.shnu.edu.cn (M.L.); 1000512181@smail.shnu.edu.cn (X.Z.); 1000514554@smail.shnu.edu.cn (J.W.); 2 Department of Psychology, Neuroscience and Behaviour, McMaster University, Hamilton, ON L8S 4K1, Canada; kongx17@mcmaster.ca; 3 School of Psychology, Parent Education Research Center, Zhejiang Normal University, Jinhua 321004, China; 4 Lab for Educational Big Data and Policymaking, Ministry of Education, Shanghai 200234, China; 5 The Research Base of Online Education for Shanghai Middle and Primary Schools, Shanghai 200234, China

**Keywords:** shyness, internalizing problems, sport engagement, sport participation, multi-level model

## Abstract

The relations between shyness and internalizing problems have been mainly explored at the individual level, with little known about its dynamics at the group level. This study aims to examine the mediating effect of individual-level sport engagement and the moderating effect of class-level sport participation in the relations between shyness and internalizing problems. The participants were 951 children attending primary and middle school from grade 3 to grade 7 (Mage = 11 years, 509 boys) in urban areas of China. Cross-sectional data were collected using self-report assessments. Multi-level analysis indicated that (1) shyness was positively associated with internalizing problems; (2) sport engagement partially mediated the relations between shyness and internalizing problems; and (3) class sport participation was a cross-level moderator in the mediating relations between shyness, sport engagement, and internalizing problems. Shy children in classes with a higher level of sport participation tend to have less sport engagement and more internalizing problems than those in classes with a lower level of sport participation. These findings illuminate implications from a multi-level perspective for shy children’s adjustment in a Chinese context. The well-being of shy children could be improved by intervening in sport activity, addressing both individual engagement and group dynamics, such as class participation.

## 1. Introduction

Shyness refers to excessive wariness, discomfort, and internal anxiety in contexts of social novelty or when perceived social evaluation is present [1]. Shyness is a temperamental trait that is considered moderately inherited and biologically based, demonstrating relative stability throughout personality development [2]. According to the social motivational perspective, social engagement arises from the interplay between two opposing motivational tendencies, social approach and social avoidance [3]. Shyness reflects the underlying approach–avoidance conflict, whereby the desire for engaging with their peers (i.e., high social approach motivation) is simultaneously inhibited by social fear and anxiety (i.e., high social avoidance motivation). This inhibition contributes to the tendency of shy children to withdraw from social interactions and have fewer opportunities to engage with peers, which may lead to negative adjustment outcomes [4]. There is growing evidence indicating shyness is associated with various adjustment difficulties, particularly internalizing problems, such as loneliness, depression, social anxiety, and higher negative affect [5,6,7]. Research has shown that sport engagement and participation are associated with a variety of positive psychosocial outcomes [8,9]. Class-centered physical education is the primary way for school-aged children to engage in sports activities in the context of Chinese culture [10]. As an important form of team sports activities, physical education class can provide a structured and supportive environment that fosters the development of individual competencies, the formation of social connections, and the expansion of support networks [11]. Thus, participation in and engagement with team sports activities holds the potential to mitigate internalizing problems in shy children. Although extensive research provides evidence linking shyness to internalizing problems, the internal mechanisms that may underlie (i.e., mediators) or modulate (i.e., moderators) these links remain under-investigated. Meanwhile, previous studies on shy children mainly focused on the individual level, with less attention given to the group level influences. Accordingly, the present study aims to fill the gap by exploring the relations between shyness, sport engagement, class sport participation, and internalizing problems among children in mainland China.

### 1.1. Shyness and Internalizing Problems in China

Shy children tend to withdraw from social interactions, increasing the likelihood of experiencing internalizing problems [12]. In the present study, we particularly focused on loneliness, depression, and social anxiety as indices of internalizing problems, which have a variety of negative consequences for both physical and mental health [13,14,15].

Peer relationships and friendships are important sources of emotional and social support [16]. Children who interact less with their peers, such as those who are shy, may be at a higher risk of developing emotional problems than their more outgoing counterparts [17]. Shy children often feel uncomfortable in social situations, making it difficult to establish deep friendships with peers. As a result, they are perceived to be sad and anxious and may even be intentionally excluded by peers, which leads to loneliness [18]. Moreover, shyness can disconnect children from peer play, contributing to feelings of loneliness [19]. It has also been reported that low levels of peer acceptance and approval in shy children result in higher levels of loneliness [20]. Empirical support from the Chinese sample has shown that shyness is associated with feelings of loneliness [21,22,23,24,25]. For example, Tan et al. found Chinese children with greater level of shyness were not conducive to developing close relationships, resulting in insufficient social support and greater feelings of loneliness [26].

Similarly, shyness interferes with the healthy development of satisfying interpersonal relationships in children. Interpersonal failures and concomitant anxiety can produce negative affect, which may cause an increase in depression [27]. Meanwhile, shy children may develop negative self-perceptions and other psychological problems such as depression when they realize their difficulties in social situations [28]. Numerous studies have shown that shyness is linked to depression [6,29,30,31,32]. 

According to the cognitive model of social anxiety, anxiety arises from the negative cognitive processing bias towards self and external information [33]. Shy children, who often focus on themselves with fear of negative judgment, are particularly prone to experiencing social anxiety [28]. Furthermore, shy individuals tend to have relatively pessimistic perceptions of their social performance, influenced by previous negative social experiences [34], which consequently leads to the development of social anxiety [35]. Previous studies have also shown that shyness is related to social anxiety in China [6,36,37]. For example, Ran et al. found that shy individuals were more likely to have low self-esteem, resulting in high social anxiety [38]. 

Research has also highlighted differences in the interpretation and implications of shyness across cultural contexts [39]. In Western societies, where assertiveness and expressiveness are strongly encouraged [40], shyness is likely to be perceived as socially immature and inappropriate [7]. However, the definition and development of shyness in China is not exactly the same as in the Western context. On the one hand, shyness from the Chinese perspective involves self-conscious avoidance of public attention and social restraint in behavior that presents as modesty and maintains social harmony, in line with traditional Chinese culture and the collectivist orientation [37,41]. On the other hand, in China, which has experienced large-scale economic and social reforms in the past 30 years, the evaluation and perception of shyness have undergone great changes from positive to negative. Specifically, in the traditional Chinese culture, shyness is often valued as a positive behavioral trait that reflects social maturity, mastery, and understanding [42]. Now, new behavioral qualities such as initiative-taking and self-expression are increasingly appreciated by individuals in the competitive urban environment [43]. Thus, shyness may be perceived as a negative quality in the current social evaluation system and has been proven to be increasingly associated with the internalizing dimension of adjustment difficulties among children and adolescents in urban China [36,44,45]. In summary, shy children’s internalizing problems deserve more attention.

### 1.2. Shyness and Internalizing Problems: Mediating Role of Sport Engagement

As mentioned above, shyness is directly related to internalizing problems, such as loneliness, depression, and social anxiety. In addition to the direct relations, the present study focused on the underlying process from shyness to these internalizing problems. Ryan and Deci’s self-determination theory [46] suggests that shy children often experience diminished feelings of competence, autonomy, and connectedness with peers, which, in turn, contributes to the development of internalizing problems like anxiety and depression. These basic human behavioral needs, particularly the sense of connection with others, coincide with the concept of sport engagement proposed by positive psychologists [47]. Ramey et al. proposed that sport engagement could be conceptualized as comprising three elements: (a) an affective component, including positive and negative responses (e.g., enjoyment, excitement, and stress) to a sport activity; (b) a cognitive component, including thoughtfulness and knowledge about the sport activity; and (c) a relational component indicating the connection to others or group (e.g., relatedness, belongingness, etc.) [47]. Drawing upon this perspective, this study proposed that sport engagement would mediate the relations between shyness and internalizing problems.

Shy children may avoid sport engagement. They were less likely to list sports activity as their primary activity as compared to other activities [48] and reported less involvement in sports activities than their more sociable peers [49]. From a motivational perspective, shy students may feel anxious or uncomfortable [7], which can lead to a lack of interest or engagement in team sports activities. In fact, there is some evidence to suggest that shy children are less likely to be involved in sports activities [48,50]. For example, Miller suggested that shy children may hesitate to become involved in team sports because of fear of negative evaluation from peers and coaches [51].

Furthermore, lack of sport engagement may be related to shy children’s internalizing problems. Positive sport engagement (e.g., enjoyment, competence, and relatedness) may be uniquely important in promoting well-being [9], as it enhances peer relations, increases self-esteem, fosters a sense of security, and reduces anxiety [52]. On the contrary, shy children tend to be more concerned with others’ evaluations [1], leading to less enjoyment and more negative experiences during sports activities. This preoccupation negatively impacts their happiness and increases their likelihood of developing internalizing problems. In fact, previous studies have explored the role of sport engagement in internalizing problems [53,54]. For example, Fletcher et al. found that sport engagement was related to lower social anxiety in elementary school children [53]. Page et al. found that children aged 6 to 11 years with higher sport activity engagement reported lower scores on loneliness [55]. Moreover, a longitudinal study showed that sport engagement was related to lowered depression across two years (from 10 to 12 years of age) [56]. Taken together, shy children could experience internalizing problems through less sport engagement. 

### 1.3. Moderating Role of Class Sport Participation

According to ecological systems theory [57], the class, as an important part of the micro-system, is a direct environment for individual activities and social interaction. Sport participation, as an aspect of the class environment, is also critical for the establishment of peer relationships and the shaping of their own behavior, which has a profound impact on children’s mental health and socialization [58]. Class sport participation refers to an organized, class-centered physical activity that is characterized by regular time schedules, specified locations, coach supervision, and a group setting and usually includes goals for performance or skill development [59]. A growing body of research has demonstrated that class sport participation is negatively associated with internalizing problems among samples of general individuals or non-shy children [8,60]. However, for shy children with social withdrawal, there are different explanations. Salmivalli’s healthy context paradox may provide new insight into the relation between class sport participation and internalizing problems [61]. This paradox proposes that an improved social environment can be detrimental for some children. For example, Bellmore et al. found that the negative impact of victimization may be especially damaging in contexts where the overall level of aggression or victimization is low [62]. Their findings were interpreted in terms of social misfit and attribution theories. Specifically, being victimized in a context where very few others share this plight, one is a social misfit deviating from what is normative. In such a context, attributing the cause of victimization to oneself is likely [61]. Similarly, for shy children, they play the role of social misfits and deviants in a class with more active sport participation, and they then believe they possess personal inadequacies due to the negative self-attribution tendency [63,64]. Thus, they may suffer more negative impacts from sport participation. Therefore, we hypothesized that shy children in high-class-sport-participation environments would develop more internalizing problems than shy children in low-class-sport-participation environments.

In addition, class sport participation is also reasonable as a moderator in the relation between shyness and sport engagement. Shy children often undergo feelings of self-consciousness, worry, and anxiety when engaging in high social interactions [7]. For example, in group play activities, shy children may behave by hovering, onlooking, and avoiding approaching [3,65]. When shy children partake in class sports activities, they may derive less enjoyment from the experience, which means less sport engagement based on the affective component of sport engagement [48]. Meanwhile, shy children are less likely to be included in class sports activities, which may lead to less bonding with other participants in class sports, meaning less relational sport engagement [51]. Therefore, we hypothesized that shy children in high-class-sport-participation environments would have less sport engagement than shy children in low-class-sport-participation environments.

### 1.4. The Present Study

This study aimed to evaluate a model linking shyness, sport engagement, class sport participation, and internalizing problems among Chinese children. Based on the relevant theories and empirical literature, the following hypotheses were forwarded: (a) shyness would be positively related to internalizing problems (depression, loneliness, and social anxiety); (b) sport engagement would mediate the relations between shyness and internalizing problems; and (c) the relations between shyness and internalizing problems as well as shyness and sport engagement would be moderated by class sport participation. It should be mentioned that the amount of time spent with peers develops steadily from middle childhood through to early adolescence [66], and peers become increasingly significant in children’s daily lives throughout this developmental stage [67]. Consequently, withdrawing from social interactions may be considered as particularly unfavorable and undesirable during this stage. In the context of traditional culture in China, social interactions have become increasingly crucial; disengagement from social settings could potentially lead to increased distress for socially withdrawn individuals, such as shy children [25]. Accordingly, we focused on children in middle childhood and early adolescence.

## 2. Materials and Methods

### 2.1. Participants

The participants were N = 951 students (509 boys, age range = 8.75 to 13.34 years old, Mage = 11.29 years, SD = 1.47 years) in grades 3 to 7 from public primary and middle schools in the urban areas of Shanghai, China. The participants included 181 third graders (Mage = 9.38 years, SD = 0.49 years), 240 fourth graders (Mage = 10.26 years, SD = 0.46 years), 169 fifth graders (Mage = 11.30 years, SD = 0.47 years), 180 sixth graders (Mage = 12.31 years, SD = 0.46 years), and 181 seventh graders (Mage = 13.41 years, SD = 0.49 years), respectively. The sample was drawn from 21 classes in total, with an average of approximately 45 students per class. All children in this sample were of the Han ethnicity, which is the predominant ethnic group in China.

### 2.2. Measures

#### 2.2.1. Shyness

The Chinese version of the Children’s Shyness Questionnaire (CSQ) was used to measure shyness [1], which consists of 19 items related to shyness (e.g., “I find it difficult to talk to people I don’t know”, “I feel nervous when I am with important people”, etc.). Children were asked to rate each item on a 3-point scale, ranging from 1 = no to 3 = yes. Higher average scores indicated greater levels of shyness. This measure has demonstrated good psychometric properties and reliability in previous studies with Chinese children [36], with a Cronbach’s alpha of 0.88 in the present study.

#### 2.2.2. Sport Engagement and Class Sport Participation

Sport engagement and class sport participation were assessed using the Chinese version of the Snapshot Survey of Engagement tool—Revised (The Snapshot Survey of Engagement tool—Revised was validated among Chinese individuals (*n* = 535). A confirmatory factors analysis (CFA) indicated that the data fit well, *χ* ^2^ = 163.31, *df* = 38, *p* < 0.001, CFI = 0.97, TLI = 0.95, RMSEA = 0.06, and SRMR = 0.03, and all loadings for items ranged from 0.66 to 0.89), which includes 11 items and has a reliability of α = 0.91 [68,69,70]. Nine of these items assess three aspects of sport engagement—cognitive engagement, affective engagement, and relational engagement —on a 4-point scale ranging from 1 (not at all) to 4 (a lot). For example, cognitive engagement includes items like “I really focus on sports activities when I am doing it”, affective engagement includes items like “I enjoy sports activities and have fun when I am involved”, and relational engagement includes items like “Sports activities help me connect to something greater than myself”. Higher average scores reflect greater sport engagement. The remaining two items measure sport participation by asking children how often they participate in sports activities (e.g., “How often do you participate in sports activities?”) and how long they have been involved (e.g., “How long have you been involved in sports activities?”). The responses for frequency range from 1 (achieved it just once) to 6 (several times a week), and for duration from 1 (just started) to 6 (more than 5 years) [9]. These frequency and duration scores are multiplied to create an individual sport participation score, which is then averaged to calculate the class sport participation score. Higher scores indicate greater class sport participation. 

#### 2.2.3. Depression

A 14-item measure of the Children’s Depression Inventory (CDI) was used to assess children’s depressive symptoms [71]. Each item offers three possible responses to capture the frequency and intensity of depressive feelings, such as “I feel like crying every day”, “I feel like crying most days”, and “I feel like crying once in a while”. Children were asked to choose the statement that best represented their feelings over the past two weeks. An average score was then calculated for each child, with higher scores indicating more severe depressive symptoms. This tool has been validated and shown to be reliable in previous research involving Chinese children [72], with the present study reporting a Cronbach’s alpha of 0.87.

#### 2.2.4. Social Anxiety

The Social Anxiety Scale for Children—Revised, developed by La Greca and Stone [73], was used to measure children’s social anxiety. Children completed the Chinese version of this scale, adapted by Liu et al. [74], which consists of 15 items rated on a 5-point scale ranging from 1 (never) to 5 (always). An example item is “Kids are making fun of me”. Higher average scores on this scale indicate greater levels of social anxiety. This scale has demonstrated reliability and validity in studies with Chinese students [74], with the current study reporting a Cronbach’s alpha of 0.92.

#### 2.2.5. Loneliness

Loneliness was measured using the Children’s Loneliness Scale, a self-report measure adapted from Asher [75]. This scale includes 16 items, such as “I don’t have any friends”, which are rated on a 5-point scale from 1 (not at all true) to 5 (always true). An average loneliness score was calculated for each child, with higher scores indicating greater feelings of loneliness. This measure has been validated and shown to be reliable in previous research with Chinese children [76], with the current study reporting a Cronbach’s alpha of 0.92.

### 2.3. Procedure

Children completed self-report measures that were group-administered during class time on a school day. The administration of the measures was carried out by trained researchers (i.e., graduate students). The Research Ethics Committee of Shanghai Normal University (No. 2023026) approved this study in 2021, which was carried out in compliance with the standards of the Declaration of Helsinki. Written informed consent was obtained from all students and their parents through the school beforehand. Extensive explanations of the procedure were provided during the administration. No evidence was found that the children had difficulties understanding the procedure or the items in the measures. Recruitment and data collection were all completed at the end of semester.

### 2.4. Plan of Data Analysis

Two-level hierarchical linear modeling (HLM) was conducted to examine the main effect, the mediating effect of sport engagement, and the moderating effect of class sport participation [77]. The analyses were conducted by SPSS 23.0 and Mplus 8.0. Drawing on previous studies [47,48,78,79], two latent variables were created as follows: sport engagement (cognitive engagement, affective engagement, and relational engagement) and internalizing problems (depression, loneliness, and social anxiety). Shyness, sport engagement, and internalizing problems were included as individual-level predictors, and child gender and individual sport participation were included as control variables at the individual level. Class sport participation was included as a group-level predictor, and class size and grade were included as control variables at the class level. As suggested by Enders and Tofighi [80], the selection of centering should not be based on statistical evidence but instead depends on substantive research questions. Group-mean centering is appropriate when the primary substantive interest involves a Level 1 (i.e., individual level) predictor, while grand-mean centering is suitable for interactions involving Level 2 (i.e., class level) variables. Thus, shyness was group-mean centered, and class sport participation was grand-mean centered.

## 3. Results

### 3.1. Descriptive Statistics

A MANOVA was conducted to test the effect of gender on all study variables. A significant effect of gender was found: Wilk’s λ = 0.90, F (7, 897) = 13.82, *p* < 0.001, and η^2^ = 0.10. Follow-up univariate analysis indicated that girls had higher scores on shyness and social anxiety and lower scores on individual sport participation and sport engagement (including cognitive engagement, affective engagement, and relational engagement) than boys. Detailed descriptive statistics are presented in Table 1. The magnitudes of the intercorrelations among the variables ranged from low to moderate, indicating that these variables measured different but related aspects of child adjustment. Pearson correlations among variables are presented in Table 2. 

### 3.2. Testing Cross-Level Moderated Mediation Model

**Figure 1:**
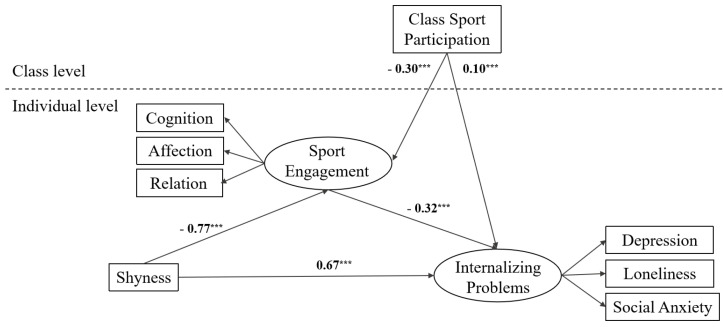
Estimates of moderated mediation model for shyness on internalizing problems. *** *p* < 0.001.

The HLM results regarding the main effects of the individual- and class-level variables and the cross-level interactions between shyness and class sport participation are presented in Table 3 and Figure 1. For within-group associations, shyness was positively associated with internalizing problems and negatively associated with sport engagement. Sport engagement was negatively associated with internalizing problems. For between-group associations, the results showed that after controlling for shyness, sport engagement, and internalizing problems, class sport participation was positively associated with internalizing problems and negatively associated with sport engagement. 

For the mediating effect, we found that shyness was negatively associated with sport engagement (*β* = −0.31, *p* < 0.001, Model 2) and, in turn, sport engagement was negatively associated with internalizing problems (*β* = −0.24, *p* < 0.001, Model 2). The positive relations between shyness and internalizing problems were significant when sport engagement entered in the same model (*β* = 0.71, *p* < 0.001, Model 2). These results suggest that sport engagement partially mediated the relations between shyness and internalizing problems.

**Figure 2:**
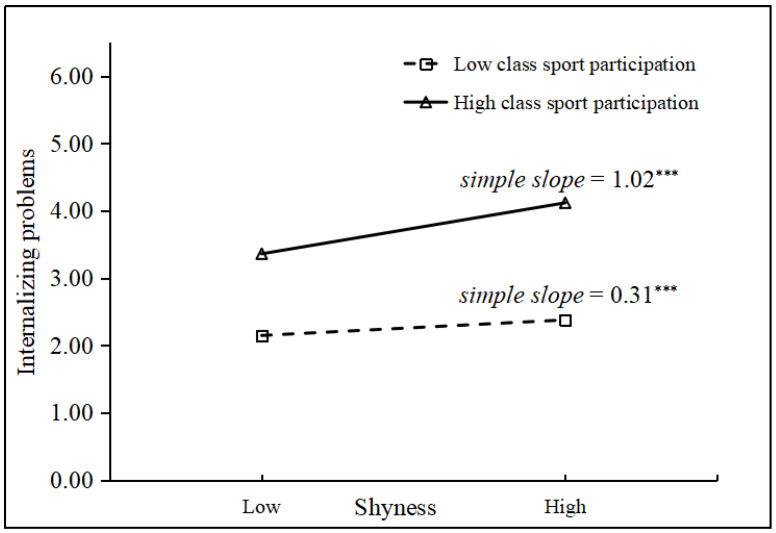
Moderating effect of class sport participation on the relations between shyness and internalizing problems. (γ = 0.09, *p* < 0.05). *** *p* < 0.001.

**Figure 3:**
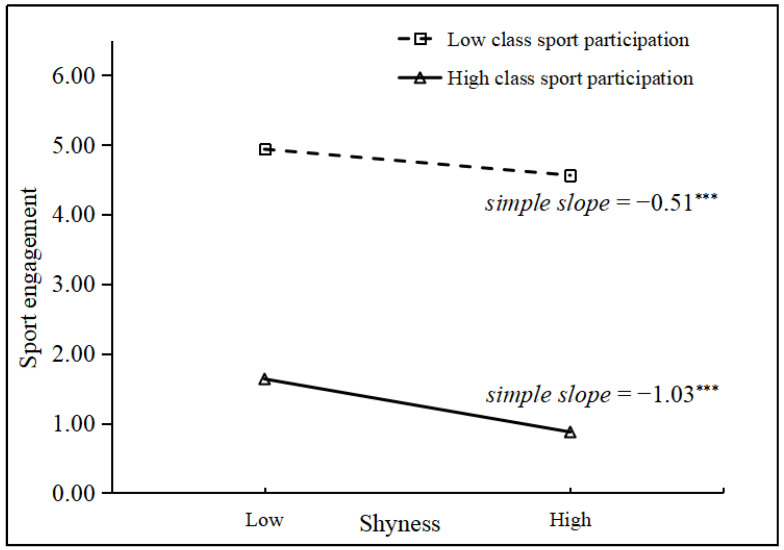
Moderating effect of class sport participation on the relations between shyness and sport engagement. (γ = −0.06, *p* < 0.05). *** *p* < 0.001.

For the moderating effects, there were significant shyness × class sport participation interactions on sport engagement (γ = −0.06, *p* < 0.05, Model 3) and internalizing problems (γ = 0.09, *p* < 0.05, Model 3). Follow-up simple slope tests were conducted to understand the moderating effects of class sport participation following the approaches suggested by Aiken and West [81]. Specifically, the associations of internalizing problems–shyness and sport engagement–shyness were plotted at a high value (1 *SD* above the mean) and a low value (1 *SD* below the mean) of class sport participation, respectively. The results are presented in Figure 2 and Figure 3. As illustrated in the figures, the associations between shyness and internalizing problems were stronger in classes with higher class sport participation (*simple slope* = 1.02, *p* < 0.001) than in classes with lower class sport participation (*simple slope* = 0.31, *p* < 0.001). In addition, the associations between shyness and sport engagement were stronger in classes with higher class sport participation (*simple slope* = −1.03, *p* < 0.001) than in classes with lower class sport participation (*simple slope* = −0.51, *p* < 0.001). In summary, higher class sport participation made children with high shyness have less sport engagement than lower class sport participation. Additionally, higher class sport participation made children with high shyness have more internalizing problems than lower class sport participation. 

## 4. Discussion

Previous studies have demonstrated that shyness is associated with internalizing problems among Chinese children [6,22]. However, the underlying mechanisms are not well understood. Therefore, we investigated a model linking shyness, sport engagement, class sport participation, and internalizing problems among Chinese children. Our findings demonstrate that sport engagement mediated the relations between shyness and internalizing problems. In addition, our result also indicated that class sport participation plays a moderating role between shyness and internalizing problems, as well as between shyness and sport engagement.

### 4.1. Shyness, Sport Engagement, and Internalizing Problems

We found evidence that shyness is negatively associated with sport engagement, which, in turn, was negatively associated with internalizing problems. To the best of our knowledge, this is the first study to demonstrate that sport engagement mediates the relations between shyness and internalizing problems among Chinese youth. Consistent with previous research, shyness has consistent links with indices of internalizing problems from early childhood through adolescence (e.g., loneliness, anxiety, and depressive symptoms) [82,83,84]. Shy children may experience heightened feelings of worry, anxiety, and rumination, especially when facing stressful situations, such as sports with peers [7]. They may not like engaging in sports due to the social interaction and communication, leading to negative thoughts about the sport and unwillingness to invest in the sport. Indeed, previous findings suggest that shy children reported less engagement in sports activities [49,85]. Sport engagement may be a salient factor for internalizing outcomes associated with shyness [86]. Individuals may experience a sense of belonging, intimate friendships, fellowship, and positive peer relationships through engaging in sports activities, all of which are linked to better mental health [60,87]. The need to belong is thought to be most important in terms of buffering against perceptions of loneliness. Intimate relationships also provide an important source of emotional support and happiness, which is beneficial to relieve depression and social anxiety [88]. Positive social contact within the context of valued relationships with people is seen as essential for greater emotional well-being [59]. In contrast, when individuals experience lower levels of sport engagement, negative thoughts may arise and feelings of connection with others may diminish, which, in turn, was associated with higher levels of internalizing problems. Take together, in our study, the less sports shy children were involved in, the more internalizing problems they experienced. 

### 4.2. Role of Class Sport Participation

We further found that class sport participation played a moderating role in the relations between shyness and internalizing problems as well as shyness and sport engagement. Specifically, a stronger sports environment in class is likely to lead to less sport engagement and more internalizing problems among shy children. Sports for children and adolescents are frequently performed within group settings such as classes [89]. Feeling of social wariness and self-consciousness may cause shy children to withdraw from opportunities to participate in class sports, resulting in their inability to enjoy and engage in sports activities. Shy children may constitute a minority group in classes where sports are popular, making them more prone to being overlooked by peers, leading to a significant psychological disparity. The team collaboration and competitive atmosphere of class sports competitions may be related to experiences of stress, which would exacerbate anxiety and tension in shy children [90]. Moreover, due to their negative attribution and poor emotion regulation ability, shy children may experience more internalizing problems [91]. In addition, according to the “healthy environment paradox” theory, despite an overall positive or healthy environment, certain individuals or groups may still experience significant difficulties, leading to negative outcomes within that context [61]. In the present study, in classes with a stronger sports environment, children who are shy and dislike sports may feel increased pressure to deal with the group activities. Due to the peer pressure and the fear of their performance in sports, shy children may disengage from sports activities, which, in turn, exacerbates the internalizing problems [92]. 

### 4.3. Limitations and Implications

The present study explored the internal mechanism and factors between shyness and internalizing problems. However, there are several limitations that must be considered. The first limitation arises from the cross-sectional, single-source research design of this study. Although we theoretically delineated the causal relations among variables, the design features increase the possibility that the results are influenced by common method variance and limit our ability to establish causal relations [93,94,95]. Thus, future research could use longitudinal or experimental approaches to examine the causal nature of these relations. Second, we used a multi-level modeling approach to analyze hierarchical data in this study, which may risk underestimating the effects. The method of Multi-level Structural Equation Modeling (MSEM) is recommended to produce more accurate estimated effects and confidence intervals [96]. Despite this, there are some challenges of using MSEM; serious convergence issues may be encountered with MSEM estimates based on fewer than 80 groups [97]. Due to its accuracy and precision in latent variable models, MSEM is still recommended for further studies, which should consider enlarging the sample size at the school level. Third, we conducted the study in urban China where the sociocultural context is different from other Chinese contexts, which limits the generalizability of the findings to other suburban or rural areas of China. Given that there are significant geographical differences in terms of social and cultural values within China, these results should be interpreted with caution when generalizing to other regions and cultures. Fourth, the measurement of sport engagement in this study was limited to the school context, without distinguishing between individual sports and team sports. Future studies could employ more detailed and effective measurements to differentiate between these types of sports activities. Finally, there are potential effects of other mediating variables that have not been discovered, such as perceived positive effects of exercise and exercise efficacy [98]. These are also considered as important variables, which are affected by individual cognition and exercise experience and deserve further attention. Future research should investigate other potential pathways to gain a more comprehensive understanding of the complex interplay between these variables.

Despite these limitations, by exploring the underlying mechanism of the relations between shyness and internalizing problems, this study extends existing research by uncovering the mediating role of sport engagement and expanding the mediation model to include the cross-level moderating effect of class sport participation. Our findings provide educational guidelines and suggestions about how to alleviate internalization problems among shy children. First, our findings have demonstrated that sport engagement is a mediator linking shyness to internalizing problems. This finding implies that wider sport engagement within the school can be essential in reducing loneliness, depression, and social anxiety among shy students. In this regard, more accessible, inclusive, and diverse sports activities should be available for students to choose from, especially for shy individuals. Second, it was found that class sport participation moderates the relations between shyness and internalizing problems as well as shyness and sport engagement. It means that the plight of shy children should be improved not only from an individual perspective but also from a group perspective such as the class. For example, teachers or coaches could build an autonomic and relaxed class sport atmosphere, which could promote shy students to establish more positive self-evaluation of their social capacity. Finally, professionals or coaches could help shy students by creating more sport programs that meet their individual needs and interests. These programs could involve sports that enhance engagement, participation, social skills, and self-esteem, and, more importantly, can be delivered in a supportive and inclusive sport environment.

## 5. Conclusions

Our study tested a multi-level moderated mediation model of sport engagement as a mediator and class sport participation as a moderator between shyness and internalizing problems among Chinese children. The results suggest that shyness is related to lower levels of sport engagement, which, in turn, is associated with lower levels of internalizing problems. A higher level of class sport participation is associated with lower sport engagement and higher internalizing problems among shy children in China. The mechanisms of these relations may inform prevention and early intervention programs for internalizing problems through the strength of class sport participation. This highlights the importance of considering individual experiences and needs within broader social contexts, rather than assuming that a positive environment guarantees positive outcomes for everyone.

## Figures and Tables

**Table 1:** Means and standard deviations of study variables.

Variable	Boys	Girls
Shyness	1.44 (0.45)	1.56 (0.41)
Cognitive engagement	3.25 (0.77)	2.87 (0.81)
Affective engagement	3.28 (0.81)	2.93 (0.92)
Relational engagement	3.20 (0.84)	2.97 (0.91)
Social anxiety	1.99 (0.84)	2.20 (0.91)
Depression	1.40 (0.33)	1.42 (0.38)
Loneliness	1.92 (0.71)	1.90 (0.75)
Individual sport participation	24.28 (11.52)	22.68 (12.08)

Note: standard deviations are in parenthesis.

**Table 2:** Pearson correlations among study variables.

Variable	1	2	3	4	5	6	7
1. Shyness	-						
2. Cognitive engagement	−0.31 **	-					
3. Affective engagement	−0.32 **	0.79 **	-				
4. Relational engagement	−0.30 **	0.74 **	0.77 **	-			
5. Depression	0.53 **	−0.37 **	−0.39 **	−0.41 **	-		
6. Loneliness	0.47 **	−0.35 **	−0.36 **	−0.40 **	0.64 **	-	
7. Social anxiety	0.74 **	−0.30 **	−0.32 **	−0.33 **	0.65 **	0.54 **	-
8. Class sport participation	0.11 **	−0.15 **	−0.11 **	−0.08 *	0.14 **	−0.02	0.14 **

Note: * *p* < 0.05. ** *p* < 0.01.

**Table 3:** Results of hypotheses testing.

Variables	Model 1	Model 2		Model 3	
	Internalizing Problems	Sport Engagement	Internalizing Problems	Sport Engagement	Internalizing Problems
***Individual level***					
Gender	−0.03 (0.03)	−0.15 *** (0.03)	−0.08 ** (0.03)	0.35 *** (0.08)	0.28 *** (0.07)
ISP	−0.09 *** (0.03)	0.26 *** (0.04)	−0.03 (0.03)	0.04 *** (0.01)	−0.01 ** (0.01)
Shyness	0.79 *** (0.02)	−0.31 *** (0.03)	0.71 *** (0.03)	−0.77 *** (0.12)	0.67 *** (0.26)
Sport engagement			−0.24 *** (0.04)		−0.32 *** (0.07)
***Class level***					
Class size				−0.01 (0.14)	−0.03 (0.09)
CSP				−0.30 *** (0.03)	0.10 *** (0.03)
Shyness × CSP				−0.06 ** (0.03)	0.09 * (0.04)

Notes. ISP = individual sport participation; CSP = class sport participation. Standard errors are in parenthesis. * *p* < 0.05. ** *p* < 0.01. *** *p* < 0.001.

## Data Availability

The data presented in this study are available on request from the corresponding author.
